# *Leptospira* spp. in Rodents and Shrews in Germany

**DOI:** 10.3390/ijerph110807562

**Published:** 2014-07-24

**Authors:** Anne Mayer-Scholl, Jens Andre Hammerl, Sabrina Schmidt, Rainer G. Ulrich, Martin Pfeffer, Dietlinde Woll, Holger C. Scholz, Astrid Thomas, Karsten Nöckler

**Affiliations:** 1 Department for Biological Safety, Federal Institute for Risk Assessment, Berlin 12277, Germany; E-Mails: Jens-Andre.Hammerl@bfr.bund.de (J.A.H.); Karsten.Noeckler@bfr.bund.de (K.N.); 2 Friedrich-Loeffler-Institut (FLI), Institute for Novel and Emerging Infectious Diseases, Greifswald-Insel Riems 17493, Germany; E-Mails: sabrina05schmidt@gmail.com (S.S.); Rainer.Ulrich@fli.bund.de (R.G.U.); 3 Institute of Animal Hygiene and Veterinary Public Health, Veterinary Faculty, University of Leipzig, Leipzig 04103, Germany; E-Mails: pfeffer@vetmed.uni-leipzig.de (M.P.); D_woll@gmx.net (D.W.); 4 Bundeswehr Institute of Microbiology, Munich 80937, Germany, E-Mails: holger1scholz@bundeswehr.org (H.C.S.); AstridThomas@bundeswehr.org (A.T.)

**Keywords:** *Leptospira* spp., leptospirosis, rodents, shrews, Germany

## Abstract

Leptospirosis is an acute, febrile disease occurring in humans and animals worldwide. *Leptospira* spp. are usually transmitted through direct or indirect contact with the urine of infected reservoir animals. Among wildlife species, rodents act as the most important reservoir for both human and animal infection. To gain a better understanding of the occurrence and distribution of pathogenic *leptospires* in rodent and shrew populations in Germany, kidney specimens of 2973 animals from 11 of the 16 federal states were examined by PCR. Rodent species captured included five murine species (family Muridae), six vole species (family Cricetidae) and six shrew species (family Soricidae). The most abundantly trapped animals were representatives of the rodent species *Apodemus flavicollis*, *Clethrionomys glareolus* and *Microtus agrestis.* Leptospiral DNA was amplified in 10% of all animals originating from eight of the 11 federal states. The highest carrier rate was found in *Microtus* spp. (13%), followed by *Apodemus* spp. (11%) and *Clethrionomys* spp. (6%). The most common *Leptospira* genomospecies determined by duplex PCR was *L. kirschneri*, followed by *L. interrogans* and *L. borgpetersenii;* all identified by single locus sequence typing (SLST). Representatives of the shrew species were also carriers of *Leptospira* spp. In 20% of *Crocidura* spp. and 6% of the *Sorex* spp. leptospiral DNA was detected. Here, only the pathogenic genomospecies *L. kirschneri* was identified.

## 1. Introduction

Leptospirosis is a zoonotic disease of global importance caused by spirochaetes belonging to the genus *Leptospira* [1]. Leptospires are usually transmitted through contact with the urine of infected animals, either directly or through exposure to contaminated water or soil. The most common portal of entry is through abrasions or cuts in the skin or via the conjunctiva [2]. The spectrum of human disease is variable and can range from subclinical infection to severe signs of multi-organ dysfunction with high case fatality rates. The severity and clinical features of the disease vary according to the leptospiral serovar as well as the age and health status of the patient [3]. To date, 20 *Leptospira* species with more than 300 serovars, grouped in 20 serogroups, have been described. On the molecular level, *Leptospira* can be divided into six environmental non-pathogenic species, nine pathogenic species and five intermediate species, for which virulence has not been demonstrated experimentally [4]. 

Leptospirosis is a notifiable disease in Germany, with the annual human incidence ranging from 0.06 to 0.2 (mean 0.08) per 100,000 between 2001 and 2013 [5]. Historically, leptospirosis in Germany was mainly associated with agricultural exposure risks [6]. As a result of mechanization, improvements in sanitation, and better control in animal reservoirs, epidemic leptospirosis in Germany disappeared in the early 1960s, although sporadic outbreaks associated with agricultural activities still occur [7]. Nowadays, recreational activities linked to freshwater exposures, e.g., swimming in lakes and canoeing, and residential exposures such as gardening and owning pets were identified as major risk factors for disease. Further, travelling abroad to other European and non-European countries was another important exposure risk factor identified for Germany [6]. This is consistent with a similar trend observed in other industrialized countries, especially among participants in adventure travel and water sports in exotic locations [8,9]. 

Among wildlife species, rodents are the most important maintenance hosts for *Leptospira* spp. and may transfer infection to livestock, companion animals and humans [3,10]. Maintenance hosts usually have no or mild symptoms after infection and excrete leptospires in the urine constantly and over long periods of time. They form an infection reservoir through continuous cycles of transmission from parents to offspring [11]. In contrast, accidental hosts such as humans can suffer severe acute forms of the disease. Leptospiruria in accidental hosts is intermittent and of short duration [12]. 

As specific rodent species may be carriers of distinct leptospiral serovars in different geographic areas, the knowledge of the prevalent serovars and the maintenance host association is essential to understand the epidemiology of the disease in a region [3].

In Germany, representatives of several genera within the order Rodentia, which include murine, vole and rat species are found. Further, shrews of different genera within the order Soricomorpha have been described [13]. Little is known about the association of the *Leptospira* spp. with the majority of these rodent and shrew species in Germany. Our objective therefore, was to gain a better understanding of the occurrence of pathogenic leptospires in rodent and shrew populations in Germany and to identify the most common *Leptospira* genomospecies. To our knowledge this is the first nationwide study in Central Europe looking at leptospiral infection in such a broad range of rodents and shrews.

## 2. Experimental Section

The sample collection was part of a multiannual study to investigate pathogen occurrence and distribution in rodents and other small mammals in Germany [14,15,16,17,18,19,20,21,22]. In the frame of the national network on rodent-borne pathogens [23] between 2002 and 2010 mice, voles and shrews were trapped in 60 different regions (defined by postal codes) located in 11 of the 16 federal states in Germany. The trapping was performed according to the routine protocols established at the different facilities. All animals were frozen and sent to the Friedrich-Loeffler-Institut for further analyses. Here, species, weight, size, and sex were determined and animals were necropsied according to the protocols of the network rodent-borne pathogens [23]. Further, the genus and species of all *Leptospira*-positive animals was confirmed by a PCR-based *cytochrome b* analysis [24]. If the genus and/or species could not be identified, the animals were not included in the subsequent analyses. The animals where the geographical origin was unclear were included in the analysis of the association between leptospiral status and rodent species, but not in the geographical presentation.

One kidney of each animal was used during the analysis. Cross contamination between animals was avoided through sequential dissection of single animals and stringent disinfection measures after each dissection. DNA was extracted from 30 mg of kidney tissue with the QIAamp DNA Mini Kit (Qiagen, Hilden, Germany). The DNA samples were distributed among three different laboratories for further analysis. As the laboratories have different routine protocols, these were used during the study. 

Laboratory A: One thousand three hundred seventy one kidneys sent to laboratory A were analysed by a duplex PCR adapted from Gravekamp *et al.* [25]. The PCR reaction was set up with 12.5 µL 2× Qiagen Multiplex PCR Mastermix, 0.5 µL primer (50 pmol/µL) and 2.5 µL template DNA. Thermal cycling was carried out with the 2720 Thermal Cycler (Applied Biosystems, Foster City, CA, USA). An initial denaturation step at 94 °C for 5 min was followed by template denaturation at 94 °C for 30 s, primer annealing at 55 °C for 30 s, a 30 s primer extension at 72 °C for a total of 35 cycles, with a subsequent final extension phase of 5 min at 72 °C.

Laboratory B: Four hundred forty seven samples were screened with a *lipl32* PCR according to the protocol by Mayer-Scholl *et al.* [26]. All positive samples were then subjected to analysis with the duplex PCR used in laboratory A [25]. 

Laboratory C: One thousand one hundred fifty five samples were tested by *lipl32* real-time PCR according to Stoddard *et al.* [27]. As in laboratory B, the less sensitive duplex PCR was used to further analyse all real-time PCR positive samples. The targets for the duplex PCR are the flagellin-encoding *flaB* gene (563 bp fragment) which only amplifies in *L. kirschneri* and the preprotein translocase-encoding *secY* gene (285 bp fragment), allowing the detection of genomospecies *L. interrogans*, *L. borgpetersenii*, *L. weilii*, *L. noguchii*, *L. santarosai* or *L. meyeri* [25]. 

Laboratories A, B, C: All samples yielding either the 285 bp fragment or both the 285 bp and 563 bp fragments were subsequently analysed by single locus sequence typing (SLST). The protocol was adapted from Slack *et al.* [28], substituting the mastermix in the protocol with the 2× Qiagen Multiplex PCR Mastermix. Due to sample size limitations only 93% of these samples could be analyzed by SLST. Samples in which the duplex PCR solely detected the 563 bp fragment were defined as genomospecies *L. kirschneri.* To confirm the results of the duplex PCR, a selection of 28 DNA samples was further tested by sequencing of the *secY* gene [29]. 

## 3. Results

### 3.1. Description of Examined Animals

**Figure 1:**
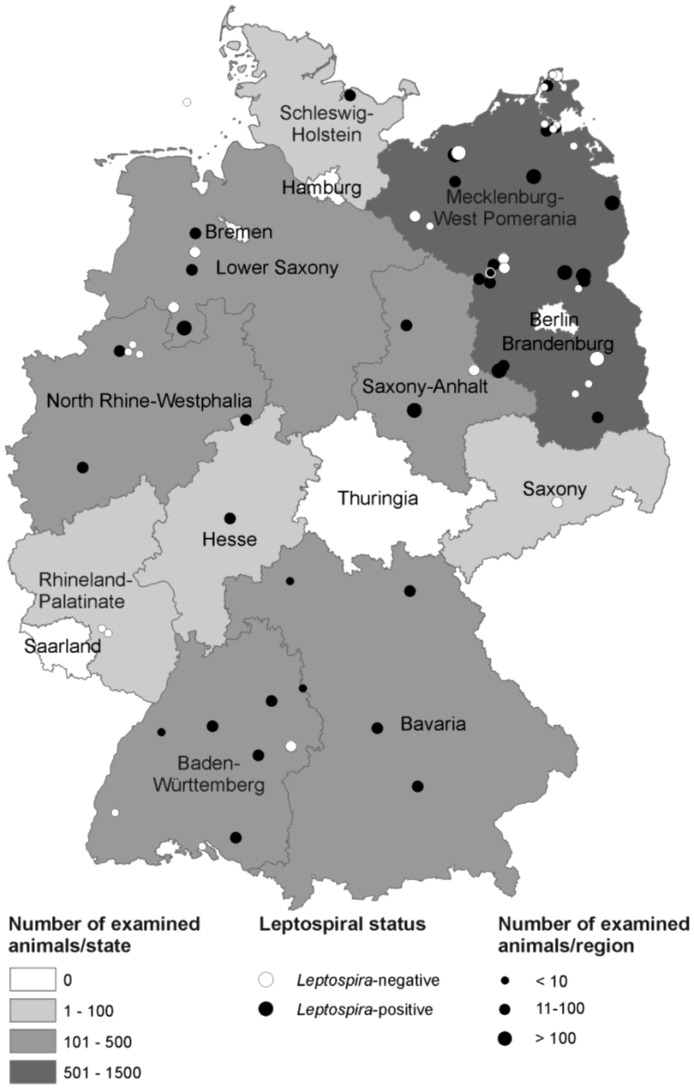
Number and leptospiral status of rodents and shrews trapped in the German federal states. The trapping site in the North Sea lies on the Island Helgoland. Several trapping regions defined by postal code include more than one trapping site. The map was created by ArcGIS 9.4 (ESRI, Redlands, CA, USA).

During 2002–2010 a total of 2973 rodents and other small mammals from 60 different regions in eleven German Federal states were collected. The majority of the animals originated from Brandenburg (36%), followed by Mecklenburg-West Pomerania (25%), Lower Saxony (13%) and North Rhine-Westphalia (8%) (Table 1). For the geographical distribution of the examined animals refer to Figure 1. 

**Table 1:** Number of animals trapped per federal state and region.

Federal State	No. of Regions	Total No. of Animals	Median (Range) Animals/Region
Brandenburg	14	1065	77 (1–241)
Baden-Württemberg	9	202	15 (1–76)
Bavaria	6	107	13 (5–45)
Mecklenburg-West Pomerania	12	758	20 (5–329)
Lower Saxony	6	377	15 (1–257)
North Rhine-Westphalia	6	226	8 (1–83)
Hesse	1	32	-
Rhineland-Palatinate	1	6	-
Saxony	1	13	-
Saxony-Anhalt	2	150	-
Schleswig-Holstein	2	34	-
Total	60	2970 *	-

Note: * The geographical origin of three animals could not be identified and therefore these animals were not included in the table.

Rodent species captured included five murine species (family Muridae): harvest mouse (*Micromys minutus*), striped field mouse (*Apodemus agrarius*), yellow-necked field mouse (*Apodemus flavicollis),* wood mouse (*Apodemus sylvaticus*), house mouse (*Mus musculus*) and six vole species (family Cricetidae): bank vole (*Clethrionomys glareolus*), water vole (*Arvicola amphibius*), field vole (*Microtus agrestis*), common vole (*Microtus arvalis*), root vole (*Microtus oeconomus*) and common pine vole (*Microtus subterraneus*). Further, six shrew species (family Soricidae) were included in the analysis: common shrew (*Sorex araneus*), Millet’s shrew (*Sorex coronatus*), pygmy shrew (*Sorex minutus*), bi-coloured white-toothed shrew (*Crocidura leucodon*), greater white-toothed shrew (*Crocidura russula*) and lesser white-toothed shrew (*Crocidura suaveolens*) (Table 2). The genus and/or species of 12 animals could not be identified. 

### 3.2. Identification of Leptospira spp. in Trapped Rodents and Shrews

Ten percent (288) of all examined rodents and small mammals were *Leptospira*-positive in the *lipl32* and/or the duplex PCR. *Leptospira*-positive rodents belonged to the genera *Arvicola* (25%), *Microtus* (13%), and *Clethrionomys* (6%) within the family Cricetidae and the genera *Apodemus* (11%) and *Mus* (6%) within the family Muridae. Animals belonging to the shrew family (Soricidae) were also identified as potential vectors for the transmission of *Leptospira* spp. In 20% of the *Crocidura* spp. and 6% of the *Sorex* spp. leptospiral DNA was detected. The regional distribution of *Leptospira*-positive animals in the different murine, vole and shrew species is shown in Figure 1.

In total, 257 samples were identified as *Leptospira*-positive by duplex PCR. In 148 (58 %) of the duplex PCR-positive samples the flagellin-encoding *flaB* gene was amplified, indicating infection with the genomospecies *L. kirschneri.* All samples further analysed by *secY* sequencing were confirmed as *L. kirschneri*. 

One hundred and nine samples where the *secY* gene (285 bp fragment) was amplified, were further analysed by SLST. As the sensitivity of the SLST analysis was much lower than the duplex PCR, only 69% of the samples showed an amplicon that could be sequenced. Of these, 68 samples (91%) were identified as *L. interrogans* and 7 (9%) as *L. borgpetersenii* (Table 2).

**Table 2:** Abundance and identification of *Leptospira* genomospecies in rodent and shrew species investigated.

Rodents and Shrews			*Leptospira*-positive Animals **		Number of Identified *Leptospira* spp. ***		
Genus	Species	No. Trapped	Total Number	Percentage	*L. kirschneri*	*L. interrogans*	*L. borgpetersenii*
***Apodemus***	*agrarius*	**190**	**23**	**12**	**19**	**0**	**0**
	*flavicollis*	**792**	**71**	**9**	**17**	**36**	**1**
	*sylvaticus*	**154**	**27**	**18**	**6**	**13**	**0**
	spp. total	**1136**	**121**	**11**	**42**	**49**	**1**
***Micromys***	*minutus*	**6**	**0**	**0**	**0**	**0**	**0**
***Mus***	*musculus*	**18**	**1**	**6**	**1**	**0**	**0**
***Arvicola***	*amphibius*	**8**	**2**	**25**	**2**	**0**	**0**
***Clethrionomys***	*glareolus*	**1016**	**66**	**6**	**38**	**18**	**0**
***Microtus***	*agrestis*	**517**	**64**	**12**	**38**	**1**	**6**
	*arvalis*	**174**	**24**	**14**	**19**	**0**	**0**
	*oeconomus*	**2**	**1**	**50**	**1**	**0**	**0**
	*subterraneus*	**4**	**0**	**0**	**0**	**0**	**0**
	spp. total	**697**	**89**	**13**	**58**	**1**	**6**
***Crocidura***	*leucodon*	**3**	**1**	**33**	**0**	**0**	**0**
	*russula*	**24**	**5**	**21**	**5**	**0**	**0**
	*suaveolens*	**3**	**0**	**0**	**0**	**0**	**0**
	spp. total	**30**	**6**	**20**	**5**	**0**	**0**
***Sorex***	*araneus*	**36**	**2**	**6**	**0**	**0**	**0**
	*coronatus*	**2**	**1**	**50**	**1**	**0**	**0**
	*minutus*	**12**	**0**	**0**	**0**	**0**	**0**
	spp. total	**50**	**3**	**6**	**1**	**0**	**0**
**Total**		2961 *	**288**	**10**	**147**	**68**	**7**

Notes: * The genus and/or species of 12 animals (all *Leptospira*-negative) could not be identified and therefore these animals were not included in the table; ** The column “*Leptospira*-positive animals” refers to the *lipl32* and/or duplex PCR results; *** Identification of genotypes was performed by duplex PCR for *L. kirschneri* and SLST for *L. interrogans* and *L. borgpetersenii*. The different sensitivities of the assays must be taken into account.

### 3.3. Detailed Description of Leptospira Detection in Selected Murine and Vole Species

As 96% of all trapped animals were representatives of the genera *Apodemus*, *Clethrionomys* or *Microtus* all subsequent data analyses focussed on these genera. 

In this study *Microtus* spp. were most often infected by *Leptospira* spp. (13%), followed by *Apodemus* spp. (11%) and *Clethrionomys glareolus* with 6% positives. 72% of the *Leptospira*-positive *Microtus* spp. belonged to the species *M. agrestis*, 27% to *M. arvalis* and 1% to *M. oeconomus*. 

Of the samples where the SLST analysis was successful, 89% of the animals of the genus *Microtus* were infected with *L. kirschneri*. The occurrence of *L. kirschneri* was equally distributed between *M. agrestis* and *M. arvalis*. Interestingly, six of the seven *L. borgpetersenii*-positive animals belonged to the species *M. agrestis* and were trapped at one site in Brandenburg. The other *L. borgpetersenii*-positive *Apodemus flavicollis* was caught at a neighbouring location in the same region. *Microtus* spp. were collected in 31 of 60 trapping regions, *Leptospira*-positive *Microtus* spp. were identified in 13 regions in seven federal states. The proportion of *Leptospira*-positive *Microtus* spp. trapped at each site ranged from 2%–100% (median 15%).

In contrast to the animals of the genus *Microtus*, bank voles (*Clethrionomys glareolus*) were less frequently infected with *Leptospira* spp. (6%) in comparison to all other rodent species examined (11%). *Leptospira* genomospecies causing infection in animals of this species were *L. kirschneri* (68%) and *L. interrogans* (32%). Bank voles were more widely distributed than *Microtus* spp. in the sample collection investigated and were found in 52 trapping regions, *Leptospira*-positive animals were trapped in 19 different regions in eight Federal States. The proportion of *Leptospira*-positive bank voles trapped at each site ranged from 6%–70% (median 12%).

Eleven percent of the tested animals of the genus *Apodemus* spp. were carriers of different *Leptospira* species. *Leptospira*-positive animals belonged to the species *A. flavicollis* (59%), *A. sylvaticus* (22%) and *A. agrarius* (19%). *Leptospira* genomospecies causing infection in this genus were *L. interrogans* (53%), *L. kirschneri* (46%) and *L. borgpetersenii* (1%). 

A difference in the distribution of trapped male and female animals was not found in the three genera and sex was not associated with detection of leptospiral DNA (data not shown). In our study, the average size of the animals did not differ significantly between infected and non-infected animals within any of the species (data not shown).

## 4. Discussion and Conclusions

To date, little is known about the occurrence and distribution of pathogenic leptospires in the most common rodent and shrew species in Germany and other European countries. Most studies have described the occurrence and identity of *Leptospira* spp. in different rat species. In Germany, Runge *et al.* [30] found leptospiral DNA in 21% of the examined Norway rats (*Rattus norvegicus*), and in France, leptospiral renal carriage was shown in 34.7% of the studied Norway rats [31]. In Poland, renal carriage in Norway rats and black rats (*Rattus rattus*) ranged between 2 and 40%, depending on the region [32]. 

Our study indicates that, albeit with a lower abundance, pathogenic leptospires are also widely distributed in murine, vole and shrew species throughout Germany. The occurrence of *Leptospira* spp. in small rodents and shrews in Europe has been investigated in earlier, less extensive studies. In a serological study in southern Germany, 8% of the 266 rodents were seropositive, primarily to the serovars Grippotyphosa and Australis [33]. Europe’s largest rodent species, the beaver (*Castor fiber*), was recently identified as a potential reservoir host for zoonotic leptospires in Germany. *L. interrogans* multi locus sequence type 24 was identified as the infecting species [34].

In a Swiss study 12.6% of the 190 examined rodents and other small mammals caught in the city of Zurich carried leptospiral DNA [35]; while in a Croatian study 7% of the 227 mice and vole species were *Leptospira*-positive in a PCR [36]. In a recent study in rodents from Lower Austria, *L. kirschneri* was detected in three *Apodemus* mice, but genomospecies *L. interrogans*, *L. borgpetersenii*, *L. weilii*, *L. noguchii*, *L. santarosai* or *L. meyeri* were found in three voles [37].

In our study *Microtus* spp. were most often carriers of leptospires (13%), followed by representatives of the genus *Apodemus* (11%). This is in contrast to a study from Croatia where house mice from an urban area showed the highest carrier rate and confirmed its role as a major reservoir in this area [36]. This difference could be due to the much lower number of house mice investigated in our study compared to the other species.

Leptospirosis is usually perceived as a zoonotic disease mainly restricted to rural areas. Yet, in a recent German study, at least 12% of the reported human leptospirosis cases were contracted in urban areas and were primarily related to residential activities, such as gardening and working on private ponds [6]. In two studies in England, *Apodemus* spp. were nearly exclusively found in residential gardens [38,39]. Also in rural areas, the wood mouse (*Apodemus sylvaticus*) is often associated with habitats close to humans [40]. As this rodent species and humans share their environment, the wood mouse may be important in the epidemiology of human leptospirosis.

In this study, *L. kirschneri* was the most prevalent *Leptospira* genomospecies. *L. kirschneri* was most common in the vole species *Microtus arvalis* and the murine species *Apodemus agrarius.* Previously published data indicate that the serovar Grippothyphosa, which belongs to the genomospecies *L. kirschneri*, is most often associated with mice of the genus *Apodemus* in Europe [3]. Interestingly, in our study *Apodemus flavicollis* and *A. sylvaticus* were more often infected with *L. interrogans.*

The public health relevance of the serovar Grippothyphosa is well documented [41]. At the beginning of the previous century, infections with the serovar Grippotyphosa were known as “mudfever” in Germany and associated with a variety of fieldwork activities [42]. In an outbreak of leptospirosis in strawberry harvesters in Germany in 2007, the common vole (*M. arvalis*) was identified as the most likely reservoir for the infection of the field workers. Leptospires isolated from the animal kidneys were identified by DNA sequencing and by monoclonal antibodies as *Leptospira kirschneri* serovar Grippotyphosa and serovar Vanderhoedeni [7].

In contrast to another study [43], the average body size, which is used in some rodents as an approximation of age and sexual maturity [44] did not differ significantly between infected and non-infected animals. This could be due to differing rodent species examined in the two studies. 

A limitation to the current study is that the serovars of the infecting leptospires were not determined. Serological classification by microagglutination is the gold standard method performed to confirm human leptospirosis in clinical cases. As a multitude of serovars are associated with a single genomospecies [3], it is difficult to infer from the molecular identification of the genomospecies to the serologically detected infecting serovar. This makes the comparison of clinical and epidemiological studies based solely on molecular methods difficult. An additional limitation was that the data origin and quality were heterogeneous, as trapping and laboratory analyses were performed according to the protocols established at the different facilities. The screening for *Leptospira* spp. in 46% of the kidney samples was performed with the duplex PCR, which is less sensitive than the *lipl32* conventional PCR [26]. The analytical sensitivity of the *lipl32* conventional PCR for rodent kidney samples is 10^2^ leptospires/mL [26]. The sensitivity of the real-time PCR is expected to be comparable, as the analytical sensitivity in spiked blood and urine samples ranged from 10^1^–10^2^ leptospires/mL [27]. The specificity of all three assays was 100% [26,27]. Therefore, the proportion of *Leptospira*-positive animals is most likely underestimated. 

In conclusion, this study was based on a very large panel of rodent species, a wide geographical coverage of Germany and a high sample size. Leptospiral DNA was detected not only in different murine and vole species, but also in some shrew species. SLST analysis revealed first insights into a host association of *Leptospira* genomospecies. 

Future studies should aim to investigate the leptospiral status of rodent species by molecular and serological assays in association with their natural habitat, environmental conditions and population dynamics in order to better understand the epidemiology of *Leptospira* in these animal populations.
